# Lead immobilization assisted by fungal decomposition of organophosphate under various pH values

**DOI:** 10.1038/s41598-019-49976-3

**Published:** 2019-09-16

**Authors:** Lin Zhang, Xinwei Song, Xiaoqing Shao, Yiling Wu, Xinyu Zhang, Shimei Wang, Jianjun Pan, Shuijin Hu, Zhen Li

**Affiliations:** 1 College of Resources and Environmental Sciences, Nanjing Agricultural University, Nanjing, Jiangsu 210095 China; 2 Jiangsu Key Laboratory for Organic Waste Utilization, Nanjing Agricultural University, Nanjing, Jiangsu 210095 China

**Keywords:** Element cycles, Environmental impact

## Abstract

Organic phosphates (OP) account for approximately 30–90% of total soil P. However, it is too stable to be utilized by plants as available P source. *Aspergillus niger* (*A. niger*) has considerable ability to secret phytase to decompose OP. Meanwhile, mineralization of lead (Pb) is efficient to achieve its remediation. This study hence investigated Pb immobilization by *A. niger* assisted decomposition of OP under variable acidic environments. *A. niger* can survive in the acidic environment as low as pH = 1.5. However, alternation of environmental pH within 3.5–6.5 significantly changed fungal phytase secretion. In particular, weakly acidic stimulation (pH of ~5.5) increased phytase activity secreted by *A. niger* to 0.075 µmol/min/mL, hence elevating P release to a maximal concentration of ~20 mg/L. After Pb addition, ATR-IR and TEM results demonstrated the formation of abundant chloropyromorphite [Pb_5_(PO_4_)_3_Cl] mineral on the surface of mycelium at pH = 5.5. Anglesite, with a higher solubility than pyromorphite, was precipitated massively in other treatments with pH lower or higher than 5.5. This study elucidated the great potential of applying OP for Pb immobilization in contaminated water.

## Introduction

Lead (Pb) is the most widespread toxic metal in the world. It presents a more serious environmental and health toxicity than any other element^1–5^. Pyromorphite [Pb_5_(PO_4_)_3_X, X = F, OH or Cl] is the most insoluble and stable form of Pb. The reaction of Pb cations and phosphate groups, causing formation of pyromorphite, is a valuable way to control the migration of Pb in contaminated environments^6–10^. Therefore, phosphates have been recognized as the successful materials to remediate Pb contamination^11–13^.

Organic phosphates (OP) in soils constitute 30–90% of total phosphorus (P), depending on soil type and land management^14–16^. Most of the OP in soil is originally from plant or microbial biomass^17^. OP is directly unavailable to plant utilization. However, it is potentially available after hydrolyzation by phosphatases^18,19^. Phytate is the most stable form of OP, accounting for up to 50% of total OP^18,20,21^. Phytase is a generic term used to describe phosphohydrolase enzymes, which can promote the sequential release of inorganic orthophosphates from phytic acid and phytates^22^. Moreover, it is widespread in nature, secreted from plants, animals or microorganisms^23^. Therefore, OP (primarily phytate) could be a potential source material for Pb remediation after phytase catalysis.

Microorganisms, the major provider of phytases, are an integral component driving soil P cycle^24^. Many phosphate-solubilizing microbes (PSM) can solubilize inorganic phosphate via secreting organic acids^25,26^ as well as releasing P from organic phosphate sources via phosphatase catalysis^27,28^. Therefore, PSM can be widely applied to increase P release from organic/inorganic phosphates^29^. Phosphate-solubilizing fungi (PSF) constitute 0.1–0.5% of total fungal populations in soil^30^ and can produce more organic acids than bacteria^31^. Moreover, PSF have been successfully utilized in industrial phytase production^32,33^. Previous studies have shown that *Aspergillus niger* (*A. niger*) is one of the most common PSF with high ability to dissolve insoluble P^34–36^. Our previous studies have demonstrated that *A. niger* enhanced P release from apatite primarily by secreting oxalic acid^37,38^. Therefore, *A. niger* has a bright future to be applied to improve the utilization of P.

Environmental pH is an important factor influencing soil quality and growth of microorganisms. During the cultivation of microorganisms, the variation of pH can cause changes of their sorption of nutrients^39^. The initial pH values of the medium can also significantly influence the acid secretion by PSM^40,41^. Zheng *et al*. observed a significant, monotonic increase in moduli of spores and hyphae over the course of incubation of *A. niger* in acidic environment^31^. Additionally, many metabolic processes of the organism are controlled by enzymes, whose catalytic responses could depend on pH^39^. However, the study regarding Pb immobilization assisted by fungal decomposition of OP under variable acidic environments is still poorly understood.

The objective of this work was to investigate the decomposition of OP and subsequent Pb immobilization by *A. niger*. Phytate was the only substrate source of P under variable acidic environments. Meanwhile, two pH adjustment treatments (e.g., initial pH adjustment treatments (EI treatments) and continuous pH adjustment treatments (EC treatments)) were set up to further study the ability of *A. niger* to adapt and change the external environment. P and Pb concentrations were analyzed by inductively coupled plasma-optical emission spectrometry (ICP-OES). High performance liquid chromatography (HPLC) was applied for the measurement of oxalic acid concentration. Then, the formed minerals during Pb immobilization were studied by using attenuated total reflectance infrared spectroscopy (ATR-IR) and transmission electron microscopy (TEM).

## Results

### Acidity of the medium and biomass of *A. niger*

Final pH values of EI treatments were shown in Table 1. The final pH value slightly decreased to 1.36 for the treatment with initial pH = 1.5, whereas the final pH values in the medium maintained around 2.0 when initial pH of 2.5–6.5. In addition, the final pH value (2.22) for the treatment with initial pH = 2.5 were significantly higher than those (2.04–2.08) of the treatments with initial pH values from 3.5 to 6.5. Therefore, *A. niger* has a strong ability to induce environmental acidity.

**Table 1:** Final pH values (Mean ± SE) of medium after five-days incubation in the EI treatments under variable acidic environments.

Treatments	1.5	2.5	3.5	4.5	5.5	6.5
Final value	1.36 ± 0.01c	2.22 ± 0.01a	2.05 ± 0.05b	2.04 ± 0.02b	2.04 ± 0.03b	2.08 ± 0.05b

**Figure 1:**
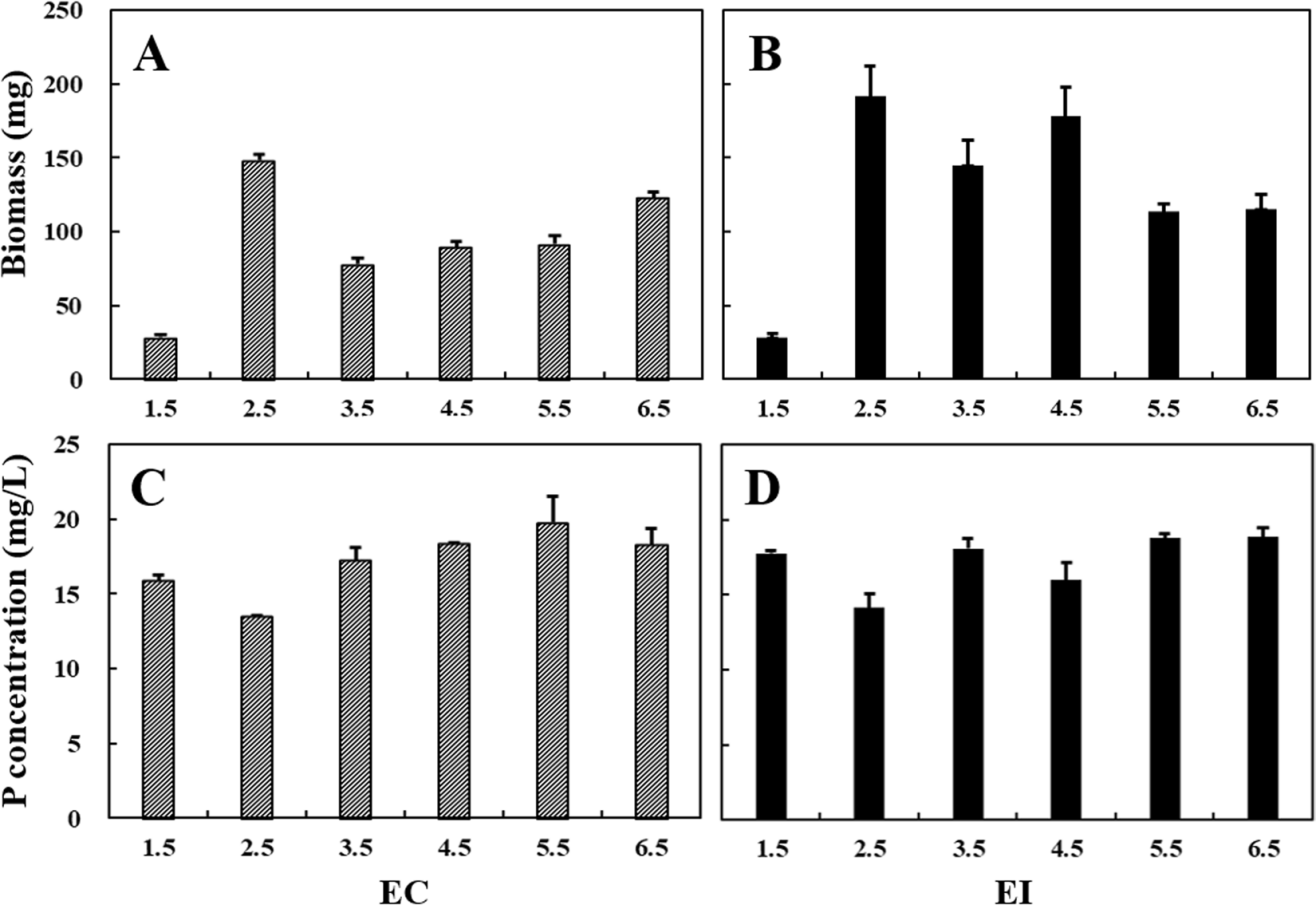
Biomass of *A. niger* and P concentrations in EC treatments (**A**,**C**) and EI treatments (**B**,**D**) under variable acidic environments. EC: continuous pH adjustment treatments, EI: initial pH adjustment treatments. Error bars represent the standard error.

Biomass can be applied to evaluate the growth of fungi in the medium. In the EC treatments, *A. niger* had the lowest biomass (28 ± 2 mg) at pH = 1.5 after five-days incubation (Fig. 1A). The biomass of *A. niger* reached the maximal value of 148 ± 5 mg as pH = 2.5. Then, the biomass (from 78 ± 4 to 123 ± 4 mg) of *A. niger* increased slowly as the pH values increased from 3.5 to 6.5.

In the EI treatments, *A. niger* had the biomass of 29 ± 2 mg at pH = 1.5, which was significantly lower than those of other treatments (Fig. 1B). This is consistent with the above EC treatments. The biomass of *A. niger* varies from 116–192 mg as pH of 2.5–6.5. In addition, the treatments with initial pH = 2.5–4.5 have the relatively high biomass for *A. niger*. For example, the biomass of *A. niger* had high values with initial pH value of either 2.5 or 4.5, i.e., 192 ± 20 and 178 ± 19 mg respectively.

### P release

There are three stages for P release in the EC treatments (Fig. 1C). As pH elevated from 1.5 to 2.5, P concentrations in the medium declined from 16 to 13 mg/L. Then, the concentrations raised to 20 mg/L when the pH values increased from 2.5 to 5.5. Compared with the treatment of pH = 5.5, a slight decline of P concentration was observed at pH = 6.5.

There was no promising trend of P release in the EI treatments, and all values fluctuated between 14–19 mg/L (Fig. 1D). For the treatments with initial pH of 2.5 and 4.5, P concentrations were declined to 14 ± 1 and 16 ± 1 mg/L, respectively, which were lower than those for other treatments.

### The oxalic acid secretion

**Figure 2:**
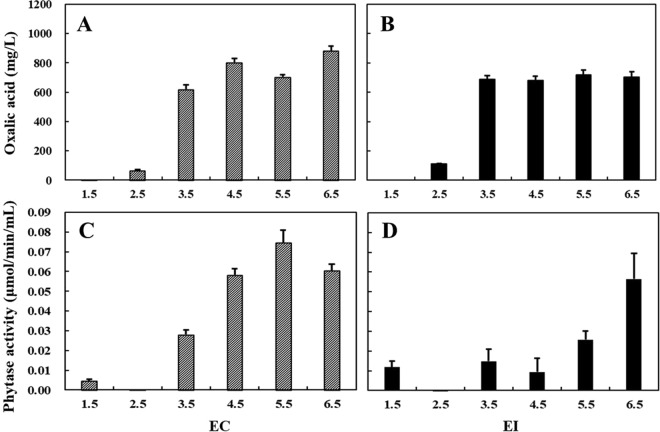
Oxalic acid concentrations and phytase activities in EC treatments (**A**,**C**) and EI treatments (**B**,**D**) under variable acidic environments. EC: continuous pH adjustment treatments, EI: initial pH adjustment treatments. Error bars represent the standard error.

Oxalic acid is the dominant organic acid secreted by *A. niger*^42^. In the EC treatments, the concentration of oxalic acid was the lowest (below the detection line) when the initial pH = 1.5 (Fig. 2A). Additionally, when the pH values of the medium increased from 2.5 to 6.5, the concentrations of oxalic acid secreted by *A. niger* elevated from 68 ± 2 to 882 ± 34 mg/L. These results confirmed that the neutral environment promoted the secretion of oxalic acid from *A. niger*.

In the EI treatments, the concentrations of oxalic acid were below the limit of detection at pH = 1.5, and there was a slight increase in oxalic acid concentration when the pH value increased to 2.5 (Fig. 2B). At pH = 3.5, the value increased six times compared with the treatment with initial pH = 2.5. However, as the pH increased from 3.5 to 6.5, oxalic acid concentrations secreted by *A. niger* had no significant difference.

### Phytase activity

In the EC treatments, phytase activities at the initial pH values of 1.5 and 2.5 are extremely low (<0.005 µmol/min/mL) (Fig. 2C), consistent with the above results of biomass and oxalic acid concentration (Figs 1A, 2A). However, the activity of phytase increased substantially as the pH value increased from 3.5 to 5.5. The phytase activity was 0.075 µmol/min/mL when pH = 5.5, which was the maximum value in all the EC treatments. Then, it decreased to 0.060 µmol/min/mL with pH value elevated to 6.5. Therefore, phytase activity increased to a high level when the medium pH near neutral (Fig. 2C), which suggests that delicate pH changes may cause considerable variation of phytase activity.

The variation trend of phytase activities in the EI treatments was different from that in the EC treatments (Fig. 2D). With the initial pH values of 1.5–3.5, the activities of phytase were all at low levels, i.e., <0.020 µmol/min/mL. It then gradually increased during pH values elevated from 4.5 to 6.5, and finally reached a maximum value (0.056 µmol/min/mL) at pH of 6.5. The value was significantly lower than those of the EC treatments as initial pH of 3.5–5.5.

### Pb^2+^ removal in the medium

**Figure 3:**
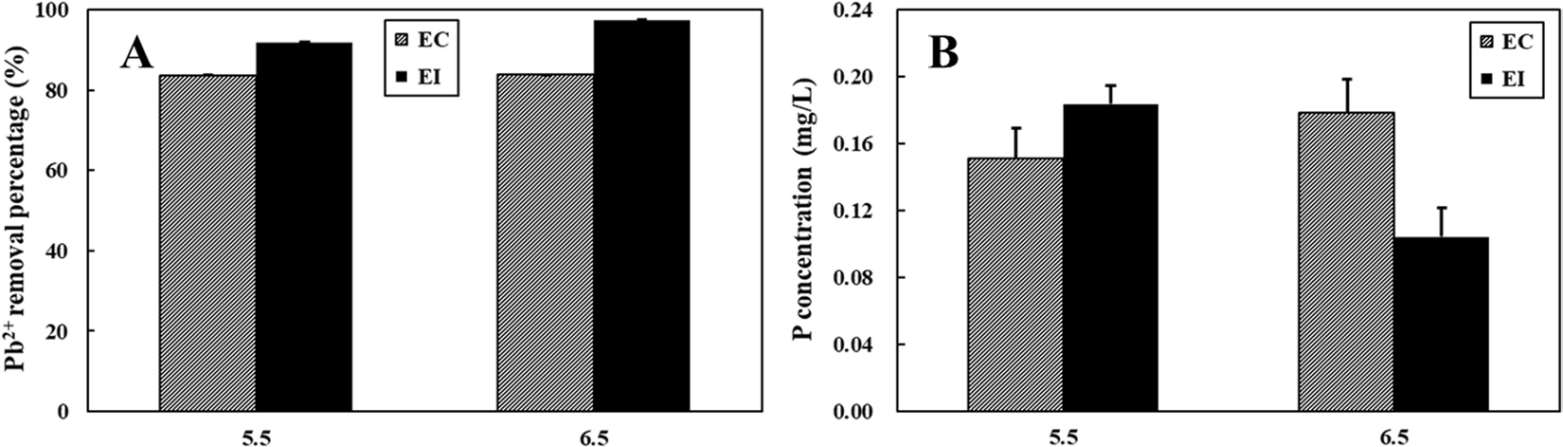
Pb^2+^ removal percentage and P concentrations in the medium after five-days incubation at pH 5.5 and 6.5. EC: continuous pH adjustment treatments, EI: initial pH adjustment treatments. Error bars represent the standard error.

The removal of Pb^2+^ by P source was efficient and widely accepted. In the EC and EI treatments with Pb addition, *A. niger* was able to remove >80% Pb^2+^ from media with addition of Pb(NO_3_)_2_ (initial Pb^2+^ concentration = 500 mg/L) and calcium phytate when pH = 5.5 and 6.5 (Fig. 3A). EI treatments showed a higher Pb^2+^ removal percentage (>92%) than EC treatments (~84%), probably due to the formation of anglesite via the reaction between SO_4_^2+^ in PVK and Pb^2+^ ^11^. Moreover, it indicated that Pb^2+^ removal percentages for EI treatments were significantly enhanced from ~92% to ~97% with the increase of initial pH.

In the EC treatments after Pb addition, the P concentration of the medium was 0.15 mg/L at pH = 5.5 (Fig. 3B). Then, it has a slight rising trend (P from 0.15 to 0.18 mg/L) as the pH increased from 5.5 to 6.5, respectively. There was no significant difference between pH = 5.5 and pH = 6.5 (*P* > 0.05). However, in the EI treatments, the highest P concentration was present at pH = 5.5 (0.18 mg/L), which was almost doubled compared with the value at pH = 6.5 (Fig. 3B).

### ATR-IR analysis

**Figure 4:**
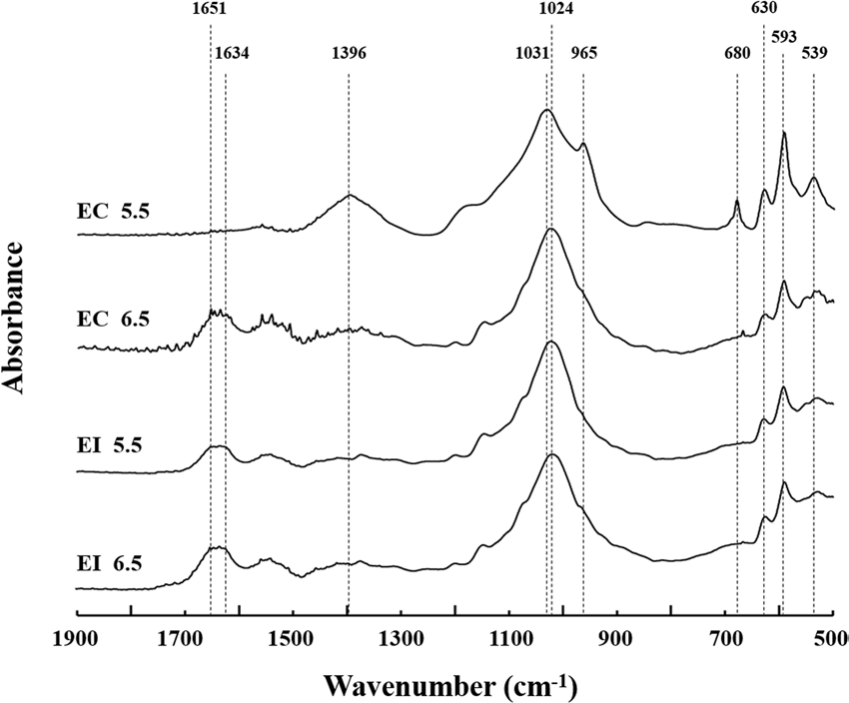
ATR-IR spectra (500–1900 cm^−1^) of the precipitates collected from the four treatments with high phytase activities, i.e., pH_EI_ = 5.5, pH_EI_ = 6.5, pH_EC_ = 5.5, and pH_EC_ = 6.5. EC: continuous pH adjustment treatments, EI: initial pH adjustment treatments.

The peaks of ATR-IR spectra located at ~593 and 630 cm^−1^ can be both assigned to S-O vibration of sulfate, which were present in all treatments^43,44^. For pH_EC_ = 5.5, the ~539 cm^−1^ peak was attributed to the *ν*_4_ mode of the PO_4_ group^45^. Compared with other three treatments, the ~593 and 630 cm^−1^ peaks at pH_EC_ = 5.5 were stronger (Fig. 4). The peak at ~965 cm^−1^ and ~1031 cm^−1^ respectively represented *ν*_1_ (PO_4_^3−^) and *ν*_3_ (PO_4_^3−^)^46,47^, which were consistent with our previous research^48^. Moreover, the intensity of the ~965 cm^−1^ peak was substantially elevated at pH_EC_ = 5.5 (Fig. 4). Besides, the representative peaks at ~539, 965, and 1031 cm^−1^ indicated that pyromorphite was formed in the EC treatments at pH = 5.5. Compared to other treatments, the new peaks at 680 and ~1396 cm^−1^ were two typical peaks for pH_EC_ = 5.5, which are represented C-O vibration in carbonates^38,49^. The presence of these peaks may indicate the formation of Pb carbonate after Pb^2+^ adsorption^43^.

The peaks at 539, 680, 965, 1031 and 1396 cm^−1^ disappeared in the EC treatments when the pH value increased from 5.5 to 6.5, confirming that no carbonate and pyromorphite were formed (Fig. 4). The most evident peak appeared at 1024 cm^−1^ represented *ν*_3_ (PO_4_^3−^) band^50,51^. Specifically, asymmetric stretching vibrations *ν*_as_ (COO^−^) appeared between 1634 and 1651 cm^−1 ^^52^. For EI treatments at pH = 5.5 and 6.5, their spectra had the similar peaks as those of pH_EC_ = 6.5.

### TEM analysis

**Figure 5:**
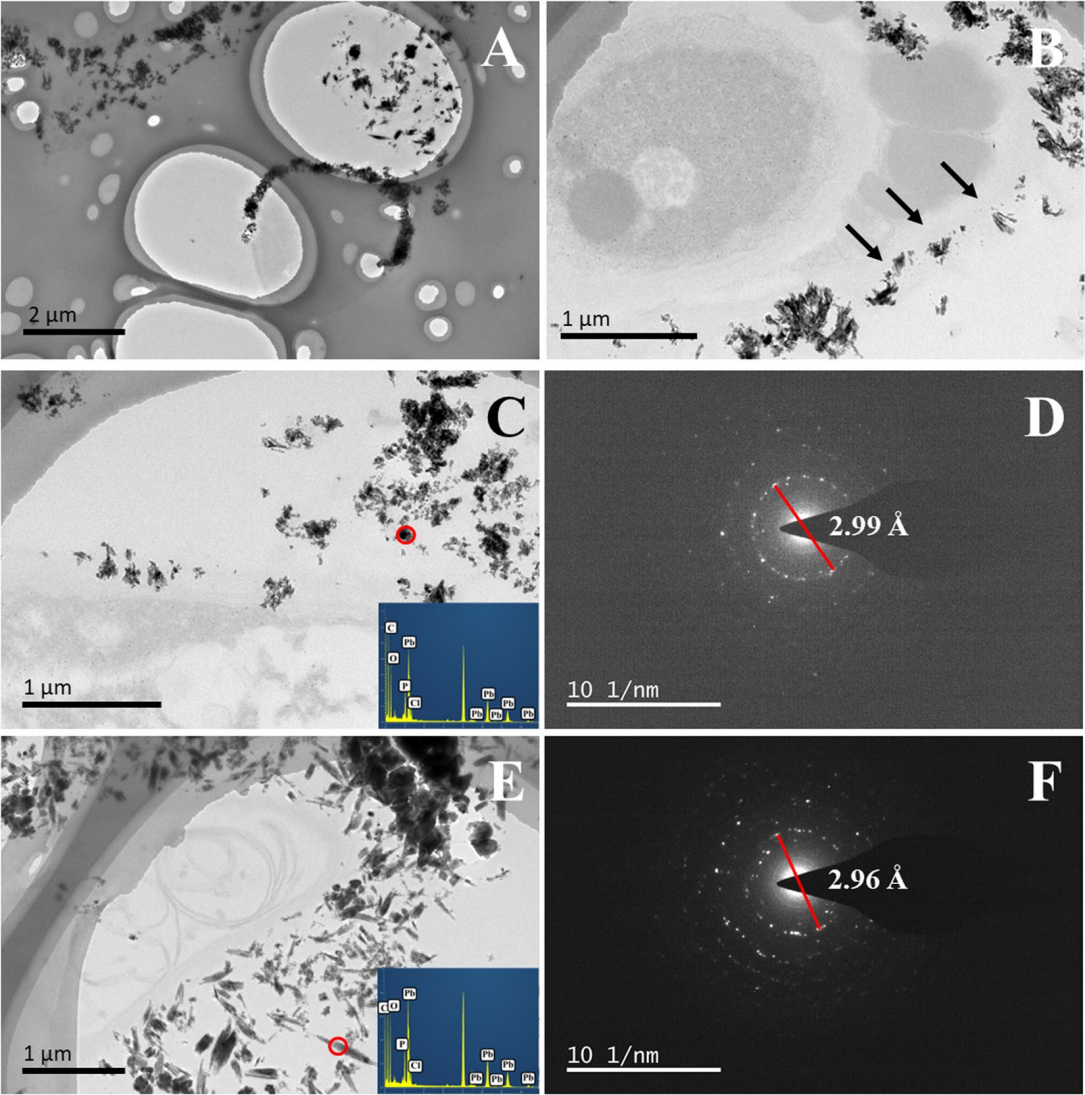
TEM imaging on mycelium (cross section) after reaction with Pb^2+^ and calcium phytate at pH_EC_ = 5.5 (EC: continuous pH adjustment treatments). Images (**A**,**B**) show that the location of the minerals around the mycelium. The arrows point to the boundary of cell wall of the mycelium in image (**B**). Images (**C**,**E**) show that the morphology of the mineralized products. The EDS spectrum of the circled area is shown in the bottom right corner. SAD patterns (circled areas) show the strong diffraction of 2.99 Å (**D**) and 2.96 Å (**F**) for pyromorphite.

Figure 5A,B showed mineralization on surface of the mycelium under TEM. TEM-EDS demonstrated identifiable Pb, P, and Cl signals in the area with dense plate-like minerals (Fig. 5C). It was then selected for detailed SAD analyses. The strongest diffraction can be identified in Fig. 5D (the calculated *d* value is 2.99 Å), which can be assigned to the (2 1 1) diffraction of pyromorphite^53^, suggesting that pyromorphite could be formed at pH_EC_ = 5.5.

Some needle-like minerals formed were observed under TEM (Fig. 5E). EDS analyses also confirmed abundant Pb, P, and Cl in these particles. Based on the SAD pattern, the characteristic (1 1 2) diffraction (*d* value = 2.96 Å) of pyromorphite can be identified in Fig. 5F. Therefore, their various shapes under TEM can be ascribed to the different orientation of crystallites.

## Discussion

*A. niger* is one of the most representative PSF, which is efficient in increasing available P. The released P enhances formation of Pb-contained minerals. Subsequently, the bioavailability (uptake of Pb^2+^ by plants or animals) of Pb^2+^ will be decreased. Meanwhile, environmental pH value is critical for microbial activities. The subsequent biomineralization is hence also significantly affected.

PSF can secrete organic acids ten times more than bacteria, lowering environmental pH as low as to 1 ~ 2^35,40^. In this study, the lowest biomass of *A. niger* was observed at pH = 1.5. This suggests that *A. niger* can survive but not prefer strongly acidic environment. *A. niger* had the highest biomass in the medium with pH of 2.5 in both EC and EI treatments (Fig. 1), whereas P concentration and phytase activity were the lowest among all the treatments (Figs 1, 2). This could be due to that *A. niger* consumes available P for their own growth, resulting in less P released into the environment^54,55^. Moreover, we also confirmed that *A. niger* is sensitive to pH alternation between 1–6. Therefore, it might be critical to control environmental pH delicately for microbial decomposition of phosphates and subsequent Pb remediation.

The low pH of the medium after incubation confirmed the secretion of abundant oxalic acid^56^. The prominent oxalate production in fungi occurs by a process called glyoxylate oxidation^57,58^. However, the trend of oxalic acid secretion is inconsistent with that of phytase. Especially, for pH_EC_ = 5.5, the high phytase activity and relatively low oxalic acid concentration were observed in solution, which suggested preference of solubilizing organic phosphates for the fungus at this pH.

*A. niger* resists Pb^2+^ toxicity via Pb mineralization (induced by releasing P from phytate) on its surface (Fig. 4). Several Pb-contained minerals are usually formed in Pb contaminated water. Anglesite (PbSO_4_) was formed in all the treatments due to the abundant SO_4_^2−^ in the medium (Fig. 4). Oxalic acid is the dominant organic acid in solution, significantly more abundant than PO_4_^3−^, and thus competing with PO_4_^3−^ during at some degree. ATR-IR results confirmed the presence of lead oxalate (PbC_2_O_4_) in several treatments. Although PbSO_4_ (K*sp* = 10^−7.7^) and PbC_2_O_4_ (cerussite, K*sp* = 10^−(9–11)^) (all with relatively high solubility) are also formed during Pb immobilization, pyromorphite (K*sp* = 10^−(72–84)^) is preferred as it is more stable^9,59–61^. In addition, abundant stable pyromorphite mineral was formed for pH_EC_ = 5.5 (Figs 4, 5). This is consistent with above phytase data, i.e., *A. niger* secreted the highest phytase at pH of 5.5, thereby increasing the probability of formation of pyromorphite^11^. Additionally, compared with common adsorptive materials (e.g., biochar and MgO^62,63^), the formation of pyromorphite via microorganisms is an environmentally friendly technique at long-term^11–13^.

**Figure 6:**
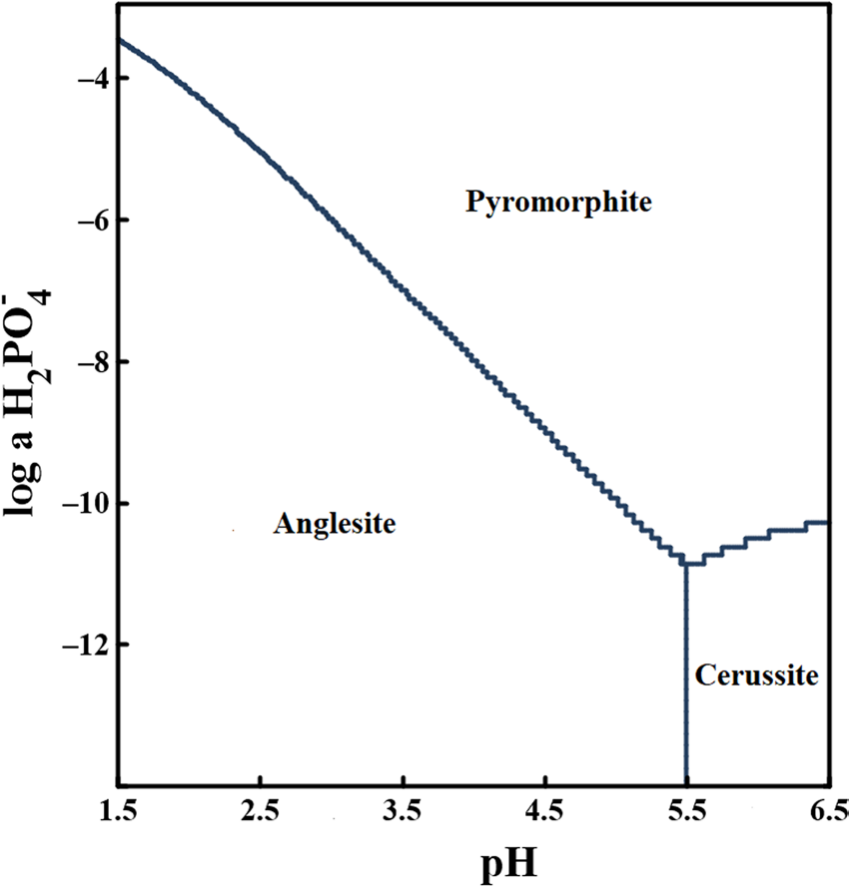
Modeling of biomineralization in the solution based on GWB software.

Geochemical modeling based on our results in water also confirms that pyromorphite, anglesite, and cerussite appear as three major species after the incubation (Fig. 6). Pyromorphite could be easily formed as long as the H_2_PO_4_^−^ concentration > 10^−11^ mM at pH = 5.5 compared to pH = 6.5. Therefore, precipitation of Pb^2+^ to pyromorphite is an effective way to immobilize Pb^2+^ and reduce Pb contamination at pH_EC_ = 5.5.

The typical limiting factor for successful remediation of heavy metals is the lack of available P. Weak acid environment promotes the decomposition of OP by fungi. It is hence an effective and efficient way for most Pb-contaminated environmental media (either for water or soil). Moreover, this study demonstrated that OP can be mineralized by microorganisms and transformed into pyromorphite mineral with addition of Pb^2+^. Therefore, this study promotes a further understanding of microorganism interactions with toxic metals and OP, as well as providing a new insight into the Pb remediation.

## Materials and Methods

### Preparation of strains and medium

*A. niger* (CGMCC No. 11544, Nanjing Agricultural University) was isolated from the soybean rhizosphere soil in Nanjing, China, and the preparation of spores has been discussed in detail in our previous research^37^.

All subsequent experiments were carried out in the modified liquid Pikovskaya (PVK) medium (the phosphate-solubilizing medium). The major ingredients of the modified PVK medium were the same as our previous study^11^, where 0.5 g TCP substituted by 0.1 g calcium phytate (Sigma Aldrich, St. Louis, MO) as P source for all the experiments.

### pH adjustment experiments

The experiments with continuous pH adjustment treatments (EC treatments, to simulate the environment with stable pH) were conducted to quantify P concentration, organic acids, and phytase activities influenced by *A. niger* in the medium under variable acidic environments. The mediums were adjusted (per 24 h) to pH values of 1.5, 2.5, 3.5, 4.5, 5.5, and 6.5 with hydrochloric acid and sodium hydroxide. Then, the solution was sterilized at 121 °C for 20 min. Finally, 1 mL spore suspension of *A. niger* (1.25 × 10^7^ spores/mL) was transferred to 100 mL modified PVK media. The media were shaken for five days at 28 °C at 180 r/min. All the treatments have three replicates.

The experiments with initial pH adjustment treatments (EI treatments, to simulate the environment with pH disturbance) were also conducted to study the ability of *A. niger* to change the various acidic environment. The experiment was a parallel experiment and was performed by setting the initial pH of media between 1.5 and 6.5 at an interval of 1.0. Most of the preparation is the same as EC treatments, except that no further pH adjustment was performed during the incubation. This parallel experiment was also continued for five days with three replicates.

After five-days incubation, the medium was filtered, and biomass of *A. niger* was weighed after drying 10 h at 80 °C. The pH values of medium were analyzed by SG98 InLab pH meter with an Expert Pro-ISM-IP67 probe (Mettler Toledo Inc.). P concentrations were analyzed after centrifugation at 4000 rpm for 30 min and by filtered through 0.22 μm membrane for Agilent 710 ICP-OES analysis. The oxalic acid concentration was analyzed by Agilent 1200 HPLC. Phytase activities were extracted by phytase extraction kit (Cominbio, Suzhou, China) and were quantified spectrophotometrically using the Spectramax i3x multi-mode microplate reader (Molecular Devices, Austria).

### Enhanced Pb immobilization by *A. niger*

EC and EI treatments with higher oxalic acid concentration and phytase activity (at pH of 5.5 and 6.5) were selected to demonstrate the influences of OP on Pb immobilization via *A. niger*. The experiment was carried out with a mixture of Pb(NO_3_)_2_ and PVK medium with initial Pb^2+^ concentration adjusted to 500 mg/L. The experiments in the fungal solution were performed with four pH adjustment treatments, i.e., pH_EC_ = 5.5, pH_EC_ = 6.5, pH_EI_ = 5.5, and pH_EI_ = 6.5. All the treatments were conducted with three replicates.

The chemical and morphological properties of precipitates after the centrifugation were determined by ATR-IR and TEM. ATR-IR technique was performed by a Nicolet iS5 Fourier transform infrared spectrometer (ThermoFisher Scientific Inc., Madison, USA) with an attenuated total reflection accessory. IR spectra were collected in the 500–1900 cm^−1^ range, with a 4 cm^−1^ resolution and 64 scans at room temperature. High resolution TEM images were collected on a FEI Tecnai G2 F20 with an accelerating voltage of 200 kV. The selected area diffraction (SAD) was also carried out under TEM.

### Modeling of Pb speciation by GWB

Module Phase2 of Geochemist’s Workbench (GWB, Version 12.0, Aqueous Solutions LLC.) was used to verify the stability of Pb minerals and the predominance of Pb chemical species after reaction. The parameters were set as shown in Table 2, based on the values of our analysis^11^.

**Table 2:** Parameters setting in Phase2 module of GWB modeling. The units of ion contents are mol/L.

Diagram species	Pb^2+^	0.0024
X axes	H^+^	10^−6.5^~10^−1.5^
Y axes	H_2_PO_4_^−^	10^−14^~10^−3^
In the presence of	SO_4_^2−^	0.005218
	Ca^2+^	0.001289
	CO_2_	−3.5
	Cl^−^	0.009157

## Data Availability

The data sets generated during this study are available from the corresponding author upon reasonable request.
